# Gene Expression Rhythms in the Mussel *Mytilus galloprovincialis* (Lam.) across an Annual Cycle

**DOI:** 10.1371/journal.pone.0018904

**Published:** 2011-05-05

**Authors:** Mohamed Banni, Alessandro Negri, Flavio Mignone, Hamadi Boussetta, Aldo Viarengo, Francesco Dondero

**Affiliations:** 1 Department of Environmental and Life Sciences, Università del Piemonte Orientale Vercelli Novara Alessandria, Alessandria, Italy; 2 Laboratory of Biochemistry and Environmental Toxicology, ISA, Sousse University, Chott-Mariem, Tunisia; 3 Department of Structural Chemistry and Inorganic Stereochemistry, University of Milan, Milan, Italy; University of North Carolina at Charlotte, United States of America

## Abstract

Seasonal environmental changes may affect the physiology of *Mytilus galloprovincialis* (Lam.), an intertidal filter-feeder bivalve occurring commonly in Mediterranean and Atlantic coastal areas. We investigated seasonal variations in relative transcript abundance of the digestive gland and the mantle (gonads) of males and females. To identify gene expression trends – in terms of relative mRNA abundance- we used a medium-density cDNA microarray (1.7 K probes) in dual-color competitive hybridization analyses. Hierarchical clustering of digestive gland microarray data showed two main branches, distinguishing profiles associated with the “hot” months (May–August) from the other months. Genes involved in chitin metabolism, associated with mussel nutrition and digestion showed higher mRNA levels during summer. Moreover, we found different gene transcriptomic patterns in the digestive glands of males when compared to females, during the four stages of mussel gonadal development. Microarray data from gonadal transcripts also displayed clear patterns during the different developmental phases respect to the resting period (stage I) with peak relative mRNA abundance at the ripe phase (stage III) for both sexes. These data showed a clear temporal pattern in transcriptomic profiles of mussels sampled over an annual cycle. Physiological response to thermal variation, food availability, and reproductive status across months may contribute to variation in relative mRNA abundance.

## Introduction

Physiological ecologists have often sought to link the internal processes of organisms with environmental factors controlling those processes in order to understand the broader distributions of populations and species. The physiological strategies that enable organisms to thrive in habitats where environmental factors vary dramatically on a month/season basis are poorly understood. The marine mussel *M. galloprovincialis* is commonly found in the Mediterranean and Atlantic Ocean coastal areas and plays a significant role in coastal ecology [1]. *M. galloprovincialis* has been extensively used in biomonitoring projects through the application of a battery of physiological and cellular markers that have yielded evidence of a stress syndrome and demonstrated the biological risk associated with polluted environments [2]. Mussels are particularly useful in this context because they inhabit regions of differential pollution status, accumulate xenobiotics, and are sessile. However, in the natural environment, the seasonal cycle is a strong determinate of invertebrate physiology (growth, reproduction, immunity) [3], [4]. Changes in environmental factors resulting from seasonal change may therefore powerfully affect the normal metabolic activities of mussels [5], [6].

In marine bivalves, studies examining physiological performance across large temporal scales have employed relatively basic proxies such as, growth rates [7], nutrient composition [8], reproductive output [9], [10] or specific gene pattern [11]. Although such work has led to great advances in our understanding of how individuals perform in response to specific environmental conditions, the next challenge is to enhance understanding of the impact of multiple environmental parameters on physiological performance of organisms across large temporal scales.

Recently, genomics-based approaches have allowed a unique view into the mechanisms underlying suites of metabolic processes. Notably, transcriptomics, the simultaneous measurement of thousands mRNAs in a biological sample, has proved to be a robust tool, enhancing our understanding of many important physiological processes in marine biosystems [5], [6]. The accessibility of new genomic resources, high-throughput molecular technologies and analytical approaches such as genome scans have made finding genes contributing to fitness variation in natural populations an increasingly feasible task [12], [13].

This work describes the first assessment involving the use of transcriptomic analysis in studying global molecular changes across an annual cycle survey in two different tissues of a wide-distributed marine ecologically relevant species. Moreover, we report that sex specific transcriptional patterns take place during gonadal developmental stages in the bivalve's tissues and that mussels' gonadal developing and maturation is driven by gene expression changes.

## Results

### Gene expression profile in *Mytilus galloprovincialis* female digestive gland during the annual cycle

**Figure 1:**
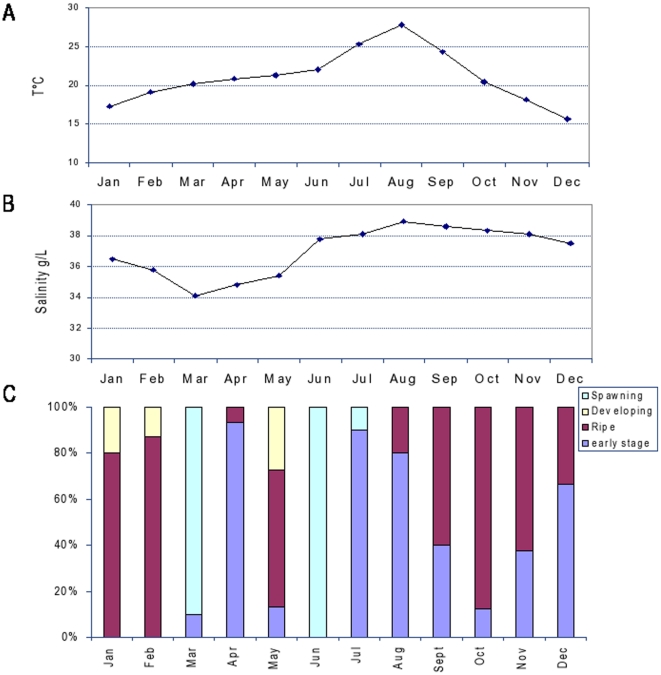
Temporal changes of mean water temperature (A), salinity (B) and gonad development (C) across an annual cycle in mussel *Mytilus galloprovincialis* (Lam.) from Bizerta lagoon (Tunisia). Note that sampling periods were as follows: March-December 2007; January–April 2008.

**Figure 2:**
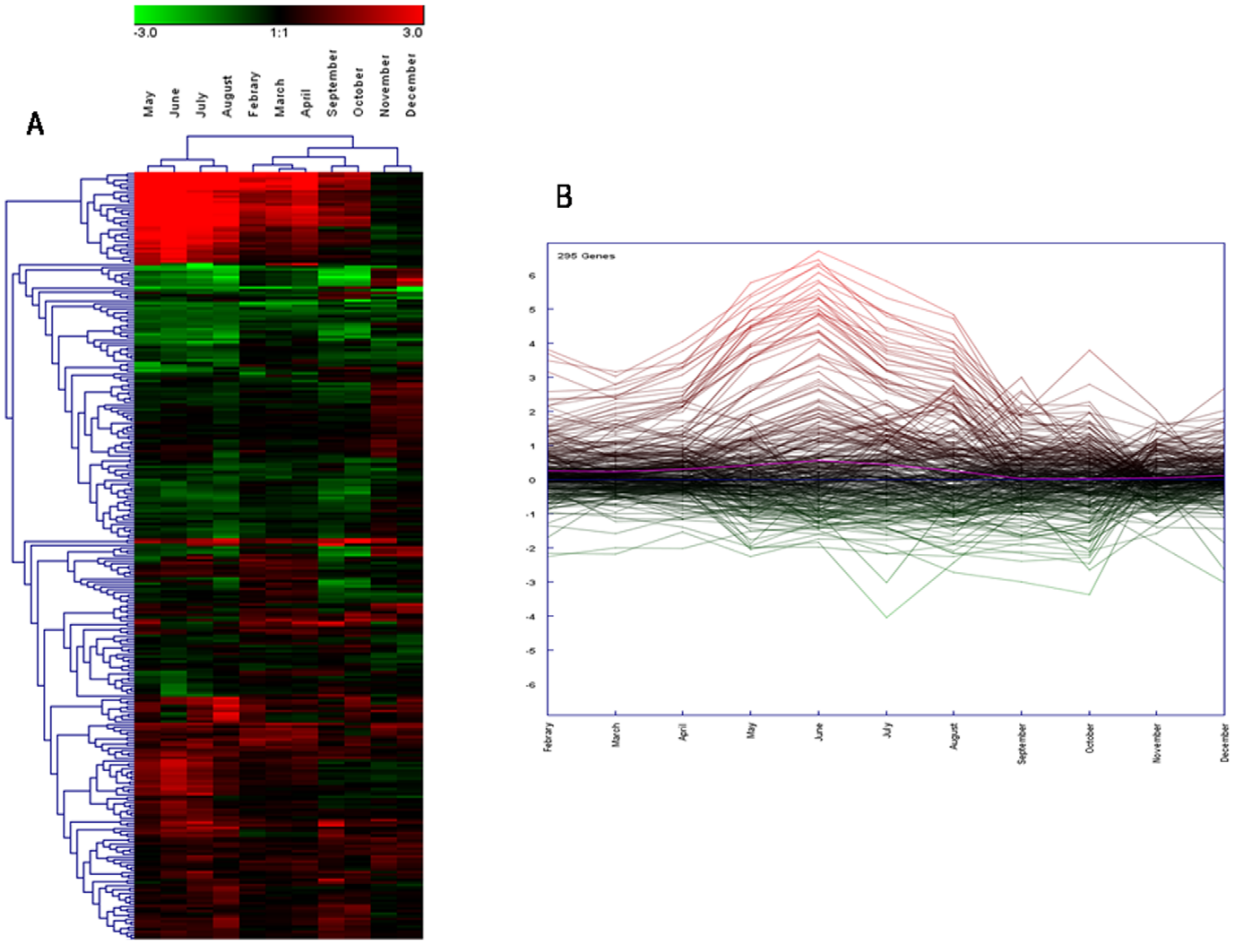
*Mytilus galloprovincialis* gene expression profiles of digestive gland tissue across the annual cycle. The heat map (A) (Pearson correlation, complete linkage algorithm) and the expression view plot (B) report the log2 relative expression level with respect to the selected reference condition (January). 295 differentially expressed genes were generated in at least one condition. Microarray data were analyzed using the Linear Mode for Microarray Analysis (LIMMA) software as described in [50]. B statistics with adjusted p value <0.05 and B>0 were used as threshold for rejection of the null hypothesis (no variation). Supporting information to Figure 2 is present on Dataset S1.

The main goal of our investigation was to use large-scale gene expression profiling to study a range of physiological pathways related to abiotic and physiological events occurring across large temporal scales (annual cycle) in a natural population of an ecologically relevant species, the mussel *Mytilus galloprovincialis* (Figure 1). Using a 1.7-K feature cDNA microarray, we generated transcriptome profiles for female digestive glands over 12 months. Microarray analysis yielded distinct patterns for 295 genes differentially expressed in at least one condition (differentially expressed genes, DEGs) (Figure 2; Dataset S1).

**Figure 3:**
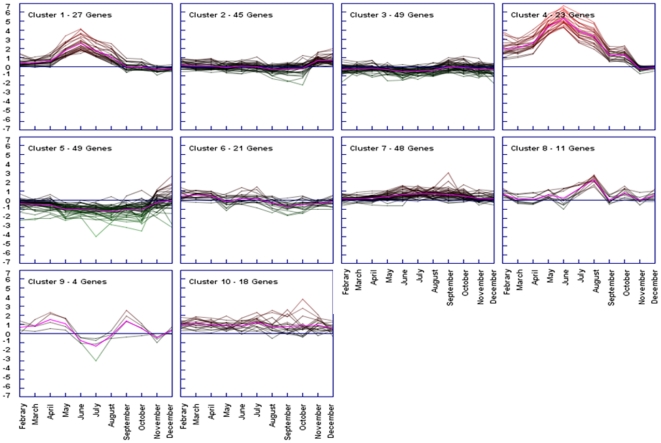
Decomposition of gene expression profile. The k-means algorithm was used for the computation of different gene expression trends in the set of 295 unique genes whose expression was modulated in female digestive gland across the annual cycle (Fig 2 and Dataset S1). K-means is an iterative procedure aimed to reduce the variance to a minimum within each cluster [55], [56]. A tendency curve (centroid) is also depicted (pink solid line). In further analysis, genes included in clusters 1 and 4 were considered together as the two groups differed only for the intensity of relative mRNA abundance. Supporting information to Figure 3 is present on Dataset S2.

To obtain more clues about the major patterns of temporal gene expression over 12 months, we performed a K-mean cluster analysis to identify distinct clusters (Figure 3; Dataset S2). Furthermore we carried out gene ontology terms enrichment analysis to identify the significant biological processes relative to each group (Table 1).

**Table 1:** GO term over-representation analysis of k-means clustered genes.

Cluster	Level	GO Term	N	Gene ID
**1–4**	4	carbohydrate metabolic process	9	AJ625361, AJ623376, AJ626213, AJ625569, AJ625778, AJ624637, AJ624093, AJ625051, AJ625276
	3	multicellular organismal development	2	AJ626213, AJ623925
	3	catabolic process	7	AJ625525, AJ623376, AJ625569, AJ625778, AJ624637, AJ624093, AJ625051
**2**	2	Cellular componenet organization	8	AJ624894, AJ623937, AJ516886, AJ516796, AJ625032, AJ625091, AJ625595, AJ626097
	4	nucleobase, nucleoside, nucleotide and nucleic acid metabolic process	4	AJ624894, AJ516428, AJ623499, AJ624625
	4	carbohydrate metabolic process	3	AJ516428, AJ516541, AJ625979
	6	protein modification process	2	AJ516541, AJ625979
	3	biosynthetic process	4	AJ623499, AJ516541, AJ624625, AJ625979
	5	ion transport	2	AJ623499, AJ624625
	3	regulation of biological process	2	AJ516886, AJ624894
	4	generation of precursor metabolites and energy	2	AJ623499, AJ624625
**3**	5	signal transduction	5	AJ625058, AJ623860, AJ624502, AJ624437, AJ625339
	3	multicellular organismal development	9	AJ626467, AJ625058, AJ624878, AJ626179, AJ624502, AJ625655, AJ624125, AJ625893, AJ624437
	2	growth	2	AJ624878, AJ626179
	4	cell differentiation	2	AJ625058, AJ624502
	3	anatomical structure development	3	AJ625058, AJ624502, AJ625655
	2	cellular component organization	3	AJ625058, AJ624502, AJ625655
	4	anatomical structure morphogenesis	3	AJ625058, AJ624502, AJ625655
**5**	4	Protein metabolic process	5	AJ625244, AJ624144, AJ624363, AJ624341, AJ516741
	3	organelle organization	3	AJ516600, AJ516663, AJ516582
	3	regulation of biological process	6	AJ516600, AJ516735, AJ516895, AJ516759, AJ625244, AJ624834
	2	growth	3	AJ516895, AJ516600, AJ516759
	4	gene expression	3	AJ516600, AJ625244, AJ623532
	2	catabolic process	4	AJ625903, AJ625142, AJ624454, AJ624363
	4	anatomical structure morphogenesis	2	AJ516600, AJ516735
	3	cellular macromolecule biosynthetic process	2	AJ516600, AJ516735
	2	multicellular organismal development	3	AJ516735, AJ516895, AJ516600
**6**	3	response to stress	2	AJ623546, AJ624410
**7**	3	catabolic process	3	AJ625903, AJ625142, AJ624454
	4	carbohydrate metabolic process	2	AJ625903, AJ625142
	2	cellular process organization	2	AJ625862, AJ624454
	6	translation	9	AJ625894, AJ624593, AJ625505, AJ624922, AJ626199, AJ624426, AJ624925, AJ624454, AJ626374
	3	regulation of biological process	2	AJ624454, AJ625862
	4	generation of precursor metabolites and energy	2	AJ625903, AJ625142
**8**	6	transcription	3	AJ624130, AJ625043, AJ625243
	3	regulation of biological process	3	AJ624130, AJ625043, AJ625243
**9**	5	cellular protein process	2	AJ624501, AJ624087
**10**	4	symbiosis, encompassing mutualismthrough parasitism	2	AJ624509, AJ623481

Gene Ontology terms enrichment analysis was carried out comparing the GO term frequency distribution into each cluster against that in the whole microarray set (hypergeometric statistics, p<0.05). Only the lowest node per branch of the hierarchical structure of the Gene Ontology that fulfills the filter condition - cut off 2 sequences- was reported. Cluster 1 and 4 were merged into a unique group as they presented the same temporal expression patterns and only differed for the intensity. Shown are: Cluster, the number of cluster obtained from k-means analysis (see Figure 3); Level, level in the GO tee of biological processes; GO Term, over-represented feature; N, number of mussel sequences associated to each GO term; Gene ID, EMBL accession number of each sequence found.

We also carried out real-time quantitative PCR (Q-PCR) to confirm and refine the relative transcription levels of 13 genes belonging to the most important clusters resulting from the K-mean cluster analysis, including heat shock protein (HSP 90), three chitinases, two metallothionein genes (mt10 and mt20), elongation factor-1, lethal giant larvae homologue-2, mam domain containing glycosylphosphatidylinositol anchor-1, matrilin, p53-like protein gene, nadh dehydrogenase subunit 5 (nd5), vitelline coat lysin m7. Microarray and Q-PCR data showed a positive correlation in, all cases, except p53-like (see Figure S1).

### Differences between male and female digestive gland transcripts across reproductive stages

Male and female digestive gland RNA extracts were evaluated during the four developmental stages of gonads according to Lowe (1982). mRNA levels evaluated by means of dual-color microarray hybridizations revealed a total of 80 (91% upregulated), 22 (36% upregulated), 49 (80% upregulated), and 32 (87% upregulated) DEGs between males and females, respectively, during the early stage, ripe, developing, and spawning stages (winter peak, from November to March) (see also Dataset S3). Functional genomics analysis based on gene ontology (GO) term enrichment statistics (hypergeometric stats, p<0.05) was carried out to identify qualitative differences between the biological processes putatively occurring in males and females (Table 2). Interestingly, differences were not related to gonadal development but to metabolic processes, in particular chitin metabolic processes and some bio-synthetic processes.

**Table 2:** GO term over-representation analysis of sex specific genes in the digestive tissue across the four stage of gonadal development.

Stage	Level	GO Term	N	Gene ID
**1**	4	carbohydrate metabolic process	7	AJ624093, AJ625778, AJ624637, AJ625361, AJ625051, AJ623376, AJ625276
	6	translation	8	AJ625361, AJ516364, AJ624922, AJ625505, AJ625894,AJ516444, AJ626199, AJ625269
	2	regulation of biological process	3	AJ516444, AJ626199, AJ625269
	3	catabolic process	6	AJ624093, AJ625778, AJ624637, AJ625051, AJ623376,AJ625525
**2**	3	primary metabolic process	5	AJ626199, AJ626374, AJ625487, AJ625525, AJ626329
	3	regulation of biological process	3	AJ626199, AJ626374, AJ625487
	2	cellular process	3	AJ625525, AJ623925, AJ626329
	3	biosynthetic process	4	AJ625525, AJ626199, AJ626374, AJ625487
**3**	4	carbohydrate metabolic process	6	AJ624093, AJ625569, AJ624637, AJ625051, AJ623376,AJ625276
	3	catabolic process	5	AJ624093, AJ625569, AJ624637, AJ625051, AJ623376
**4**	4	carbohydrate metabolic process	4	AJ625569, AJ625778, AJ623376, AJ625276
	2	cellular process	3	AJ624894, AJ625425, AJ623925
	2	cellular component organization	3	AJ623925, AJ625425, AJ624894
	3	catabolic process	4	AJ625569, AJ625778, AJ623376, AJ624894

Gene Ontology terms enrichment analysis was carried out comparing the GO term frequency distribution into each cluster against that in the whole microarray set (hypergeometric statistics, p<0.05). Only the lowest node per branch of the hierarchical structure of the Gene Ontology that fulfills the filter condition - cut off 3 sequences- was reported. Showed are: Stage, developmental stage of gonad; Level, level in the GO tree of biological processes; GO Term, over-represented feature; N, number of mussel sequences associated to each GO term; Gene ID, EMBL accession number of each sequence found.the over-represented GO terms in males versus females (hypergeometric stats, p<0.05).

### Specific gene expression fingerprint in mussel gonads during the four reproductive stages

**Figure 4:**
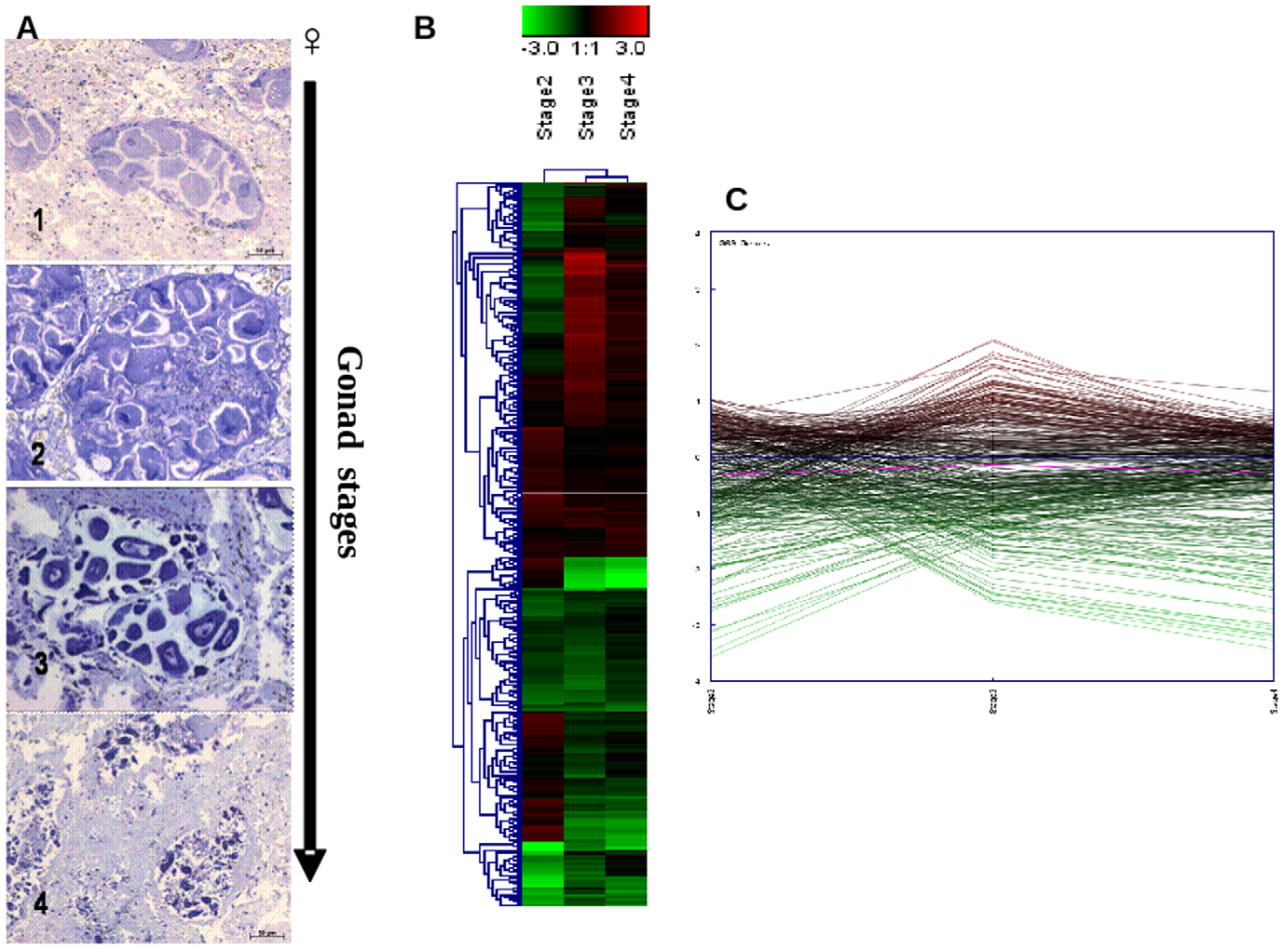
mRNA abundance patterns across female gonad maturation in mantles. Shown are representative images of the reproductive stage of female gonad (1: Early stage-November 2007, 2: Development -January 2008, 3: Ripe -February 2008, and 4: Spawning -March 2008) determined according to [46], the heat map (B) (Pearson correlation, complete linkage algorithm) and the expression view plot (C) obtained for each stage vs the reference condition (stage I). 369 differentially expressed genes in at least one condition were considered for the analysis. Supporting information to Figure 4 is present in Dataset S4.

**Figure 5:**
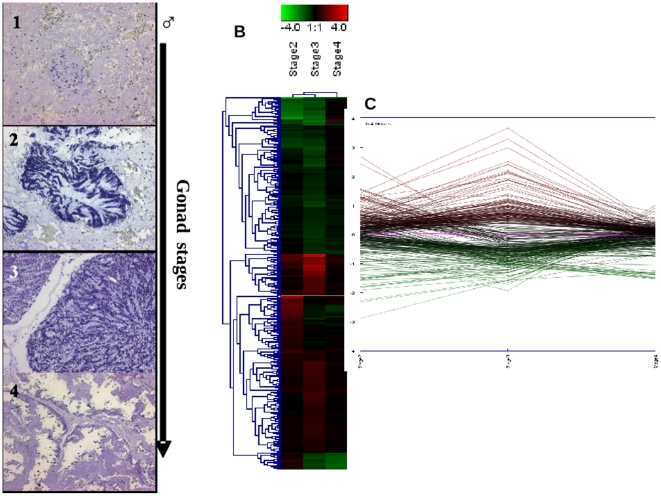
mRNA abundance patterns across male gonad maturation in mantles. Shown are representative images of the reproductive stage of male gonad (1: Early stage-November 2007, 2: Development -January 2008, 3: Ripe -February 2008, and 4: Spawning -March 2008) determined according to [46], the heat map (B) (Pearson correlation, complete linkage algorithm) and the transcription view plot (C) representing the log2 relative expression obtained for each stage vs reference condition (stage I). 354 differentially expressed genes in at least one condition were considered for the analysis. Supporting information to Figure 5 is present on Dataset S5.

To obtain clues from the gene transcription patterns in the mantle tissues during the stages of gonadal development in males and females, we also performed dual-color microarray hybridizations for these tissues across the four stages. Multivariate analysis clearly showed a distinct pattern of mRNA abundance in mantle tissues during the stages of gonadal development for both sexes. The greatest differences with respect to the reference condition (early stage) were observed at stage III (developing) (Figure 4; Dataset S4). In males, 354 DEGs -identified in at least one of the four conditions- were further analyzed. The heat-map and expression plot of male DEGs clearly showed a distinct pattern (Figure 5) that indicated an upregulation of spermatozoid maturation-related genes during stage III, including vitelline coat lysin M7, vitelline coat lysin M6 precursor and acrosomal major protein M3 (Dataset S5; Figure S3). The analysis of the 369 DEGs identified in female mantle across gonadal developmental stages showed upregulation of genes associated with mitochondrial activity, such as ATP synthase and NADH dehydrogenase subunit 5, and a pronounced down-regulation of chitin metabolism–related genes (see Figure S2 and Dataset S4).

## Discussion

### Occurrence of transcriptional oscillations in female digestive gland tissues over the annual cycle

The mussel digestive gland represents the most active metabolic organ, making it suitable for genomic profiling [14], [5]. The most pronounced pattern of gene expression deriving from the annual cycle survey was that showing a maximum activation during the hot months (relative to January temperatures) with 50 DEGs from a total of 295 (Fig. 3, clusters 1 and 4). Chitinase-related genes were markedly upregulated, as was also confirmed by the Q-PCR data (Figure S1), suggesting an evident influence on chitin-related metabolic processes.

In chitin-containing organisms, chitinases are essential for maintaining normal life cycle functions such as morphogenesis [15] or cell division and immunity [16]. In mussels and other marine invertebrates, chitinases play a role in digestion and in the control of growth and remodelling processes in a manner similar to its mammalian counterpart [17], [18]. During the hot season, Bizerta Lagoon (the sampling site) is characterized by a main peak of phytoplanktonic development that begins at the end of April and lasts for 3 months [19], [20], in June, levels of chlorophyll-a reach a maximum of 4.4 µg/l. The summer phytoplanktonic bloom is mostly characterized by diatoms [19], which increase food availability for mussels. Moreover, diatoms were recently reported to produce chitin, which plays a central role in their biology [21]. In this respect, chitinase-related activity should be considered as typical responses in bivalve mollusks during the periods of more intense feeding activity as a consequence of higher food metabolism.

We identified further trancriptomic changes associated with the acute thermal stress: K-mean cluster analysis revealed the upregulation of a set of thirteen genes involved in different macromolecule metabolic processes. Among these it could be found a general transcription factor, two translation factors (elongation factor-1 alpha and eukaryotic translation initiation factor subunit 3), a calcium dependent phospholipase, a serine protease and a heat shock protein 90 (HSP90). These data support the initiation of a metabolic activation in digestive gland which is typical of the post gametogenesis period and that should allow the accumulation of energy stores to fuel the further reproductive stages in the colder months [59].

HSPs play an important role in protection against multiple stressors, namely heat stress, toxic metals, and ionizing and UV radiation, assisting in ATP-dependent folding and stabilization of stress-damaged proteins [22], [23], [24]. Moreover, recently it has been shown that in the Pacific oyster *Crassostrea gigas* the expression of HSP90 genes is elevated after acute thermal stress [4]. Indeed, Hsp90 interacts with proteins that have already attained a high degree of tertiary structure, and appears to be involved in late-stage maturation and activation of these “client” proteins. The known client proteins include steroid hormone receptors, helixloop-helix transcription factors, tyrosine and serine/threonine kinases and tumor suppressors [25]. Therefore, an exclusive role in stress protection for the *Mytilus galloprovincialis* HSP90 -whose expression raised in June and drop thereafter (Fig. S1)- cannot be assigned without further investigations.

Another marked upregulation trend in the transcriptomic profile as revealed by the K-mean cluster analysis coincided with August. Genes associated with immune system processes, such matrilin and the p53-like protein gene were identified. Matrilin transcript abundance was confirmed by means of Q-PCR analysis (Fig. S1). Matrilin has a defensive function in zebra mussel hemocytes upon antigen stimulation [26], and a matrilin-like molecule has been reported from the freshwater snail *Biomphalaria glabrata* which confers resistance to infection with the helminth *Echinostoma caproni* [27]. A variety of cells express p53-like mRNAs, such as hemocytes of the common mussel *Mytilus spp* [28], [29], which are innate immune cells with neutrophil-like activities. The joint activation of p53 and matrilin variants in August would suggest a defense strategy adopted by mussels during the most likely infectious (e.g., from toxic dinoflagellate species) period in the annual cycle. However, the mRNA abundance trend of the p53-like gene could not be confirmed by Q-PCR which instead showed an opposite pattern (Fig. S1). It should be pointed out, however, that p53-like exists with several 5′ and 3′ splicing variants also in *Mytilus spp* [29] and therefore a further detailed investigation using specific exon probes will be required.

By contrast, Q-PCR analysis confirmed the microarray outcome for what concern the transcriptomic pattern of the metallothionein mt10 gene. As known, metallothioneins are pleiotropic proteins involved in the homeostasis of both essential and noxious heavy metals, with a role also in oxidative stress scavenging [57]. Indeed, mt10 displayed an uneven trend of over-expression with higher values in May-June-November and a drop in July-August (Dataset S1 and Fig. S1). In digestive gland of *M. galloprovincialis*, mt10 is expressed at high levels and its main function is concerned with the homeostasis of essential metals such Zn and Cu [52]. Conversely, the cognate gene mt20 (which was not represented onto the microarray) is tightly repressed in normal conditions, merely insensitive to copper, responsive to zinc, fairly inducible by mercury, dramatically by cadmium and even susceptible to oxygen reactive species [52]. In past years, this extraordinary modularity and selectivity of the mussel mt10/mt20 inducible system allowed our research group to develop a very effective tool to assess heavy metal pollution in tissues of *Mytilus spp* specimens sampled along coastal areas [30]. The finding that metallothionein mt20 mRNA abundance showed no significant changes during the investigated period but yet a down-regulation in August (Fig. S1), opens the possibility to copper fluctuations in sea water to explain the much more consistent variations observed for the cognate mt10 gene. In support to this hypothesis is that Cu could be found at relative high concentration in sediments close to the site where mussels were collected [58]. Furthermore, in view of the fact that July and August matched the post-spawning period where ovaries were almost spent (Fig. 1) and a drop of mt10 mRNA abundance was observed, it is also conceivable that mt10 gene expression variations could be linked to some relevant physiological process, viz. reproduction, thus involving a transfer of zinc (and copper) from the digestive gland to the gonad and finally to gametes. As a corollary, these findings have got an important consequence in marine biomonitoring applications because they confirm the hypothesis drawn in [30], [48] where the inconsistency of mt10 induction by heavy metals was explained with the huge variability of its basal expression level.

### Divergence in mussel digestive gland transcripts between males and females during gonadal developmental stages

In view of the ecological relevance of *Mytilus. ssp* to the marine environment, understanding their biology is important especially for basic processes such as reproduction, speciation mechanisms, and adaptation to stressors. To extend our investigation into the role of the reproductive status in males and females, we carried out a stage-by-stage comparison of the digestive gland transcripts from both sexes using a functional genomics analysis based on GO term enrichment statistics. Enrichments in the GO categories of Biological Process were identified. Interestingly, GO analysis did not highlight processes or molecular functions related to pathways specific to gonadal development but did indicate some differences for both catabolic and biosynthetic processes. In particular, according to gene expression data, the chitin metabolism appeared more pronounced in males when compared to females individuals across almost all stages (with the exception of Stage II). Some differences at biosynthetic level arose at stage I and II where a differential modulation of genes involved in protein translation and regulation of gene expression was observed respectively at stage I and II of gonadal development. The differences in the latter two stages were still dominated by the over expression of chitinases in the tissue of males. These latter findings emphasize the need to determine the sex of mussel specimens during ecological/ecotoxicological investigations that use this organism as a sentinel and the digestive gland as a target tissue.

### Gene expression during the gonadal development stages in males and females

The maturation of gametes may result from changes in both gene transcription [31] and protein translation [32] that occur during this developmental period. Numerous studies have investigated reproductive mechanisms in mollusc species at the biochemical and physiological levels; however, few have described these mechanisms at the molecular level. One report is that of [33], which characterized reproduction-specific gene expression in the marine scallop *Argopecten purpuratus* and its relationship with maturation stage and sex.

To explore how gonadal development might be linked to oscillatory patterns of relative mRNA abundance, we investigated mantle transcript variations across the four stages of gonadal development. In both sexes. The data -expressed relative to the first stage- revealed maximum change during stage III (ripe). In males, the upregulation of acrosomal major protein M3 and M6 and vitelline coat lysin M6 and M7 during the developing stage as well as the relative abundance of putative microtubule-associated protein and tubulin mRNA suggested that this phase is crucial for male gonadal development. Indeed, in mussels, coat-lysin proteins are found in sperm acrosomes and allow dissolution of the egg vitelline coat, permitting fertilization [34]. Moreover, vitelline coat lysine transcripts were recently used as molecular target to identify gender during ripe and spawning phases in mussels [35]. The tubulin gene family is important for development from gametes to hatching, involving often rapid, complex changes in the gametes and embryonic cells that are reflected in underlying changes in gene expression [36]. Moreover, [37] reported that levels of alpha- and beta-tubulin mRNA increase 25-fold around the time of transition between spermatocytes and spermatids when sperm tail synthesis is initiated in flounder. The TaqMan assay confirmed the transcription trend of the vitelline coat lysin M7 (Figure S3), making this report, the first to highlight the importance in the developing stage of genes that are key to spermatozoid maturation in male *M. galloprovincialis*.

In female mussels, the trancriptomic profile in mantle tissues clearly implicated mitochondrial-associated genes in gamete maturation during the third phase. In the same stage, a down-regulation of the chitinase variants also was identified. The mRNA abundance trend for NADH dehydrogenase subunit 5 (ND5), one of the most upregulated genes, as well as chitinase, was confirmed by means of the TaqMan Q-PCR assay (Figure S2). Mitochondria are the most abundant and prominent organelles in the early embryo [38] and are thought to be exclusively derived from the oocyte [39]. Oocyte mitochondria must support early embryonic development until the resumption of mitochondrial replication. [40] reported that during oogenesis, there is amplification in mitochondrial number in parallel with cytoplasmic volume increase. Moreover, the increase in mitochondrial number during oocyte growth is accompanied by changes in their ultrastructure [41]. [42] described a high ATP turnover, supplied by mitochondrial respiration in human mature oocytes.

The relative abundance of the mRNA of chitinase variants during the early stage of gonadal development relative to the ripe, developing, and spawning stages may be related to vitellogenesis in female tissues of scallop, *Patinopecten yessoensis* [43]. Indeed, recent work using RNA interference has suggested that in *Acanthocheilonema viteae*, a filarial nematode, expression of chitinase is associated with gender at the developmental stage [44]. [45] reported for the first time the gradual decrease of C9-chitinase mRNA during embryonic development in the oyster *C. gigas* and suggested that early developmental expression of the chitinase variant genes has to be considered as maternal in origin.

Our study provides the first description of temporal variation in gene expression patterns between sexes and with gonadal development stages in one of the most-used species in marine ecotoxicological surveys, *Mytilus galloprovincialis*. The physiological response to thermal variation, food availability, and reproductive status across months appears to contribute to the variation in gene transcription in female digestive gland tissues. Moreover, our data suggest that during the developing stage, abundance of mRNA related to gonadal maturation peaks, indicating that this stage is crucial in mussel reproduction.

## Materials and Methods

### Sample collection

Specimens of Mytilus. galloprovincialis (4–5 cm length) were collected monthly between May 2007–April 2008 from a sub-tidal mussel population located in the Bizerta Lagoon, Tunisia (Universal Transverse Mercator coordinates: Zone, 32 S; Y, 4119725.04 m N; X, 581523.57 m E). Mussels, groups of 15–20 individuals, were maintained submerged into insulated 60 L tanks containing aerated sea water collected directly from the sampling site. The animals were transported to the University laboratory within 2 h from the collection site using an insulated van. Digestive glands and mantles were further removed from mussels, washed in artificial seawater buffered with 20 mM HEPES pH 7.4, and stored accordingly to further analyzes. For transcriptomics, the tissue was kept at −20° C into a RNA-preserving solution (RNAlater, Sigma-Aldrich); for histochemistry, mantle tissues were flash frozen in liquid nitrogen and stored at −80 °C.

Water temperature and salinity were routinely assessed during each monthly sampling using standard approach.

### Sex and reproductive stage determination

Briefly, frozen slices of mantle tissue were washed in 0.05 M cacodylate buffer (pH 7.4), fixed in 1% paraformaldehyde dissolved into 0.05 M cacodylate (pH 7.4) for 30 min at 4 °C, and washed again in 0.05 M cacodylate. Samples were then dehydrated in increasing acetone concentrations at 4 °C and embedded in Technovit 7100 resin (Heraeus Kulzer, Wehrheim, Germany). Serial cross sections (2 µm) were cut using a HM350 Microm microtome (Walldorf, Germany), transferred onto glass slides, and stained using toluidine blue. Determination of reproductive stage for mussels was based on a histological evaluation of the maturation stages of gonads [46]. The reproductive cycle of *M. galloprovincialis* can be described in terms of four readily identifiable stages (early, development, ripe, and spawning). Each individual was categorized into one of these stages. 20 different animals per month were considered for the analysis.

### Microarray analysis

Total RNA was extracted from single sexed individual digestive gland pieces using acid phenol-chloroform precipitation according to [47], with the TRI-Reagent (Sigma-Aldrich). RNA was further purified by precipitation in the presence of 1.5 M LiCl_2_. The quality of each RNA preparation was verified both by UV spectroscopy and TBE agarose gel electrophoresis, in the presence of formamide as described in [48]. Competitive dual-color microarray hybridization analysis -including dye swap- was performed using the Mytarray V1.1 platform [49]. This array encompasses 3′ cDNA probes representing 1748 independent mussel sequences obtained from unbiased *M. galloprovincialis* tissues-specific cDNA libraries. cDNA fluorescence-labeled probes were obtained by the direct labeling procedure in the presence of modified cy-3 and cy5 dCTP (Perkin Elmer) [51]. Briefly, 15 µg of total RNA were primed with 0.5 µg oligodT(19)VN primer at 70°C for 10 min, then reversed transcribed at 42°C for 2 h in the presence of 400 U ReverseAid MuLV H minus reverse-transcriptase (Fermentas), and 100 µM each dATP, dTTP, dGTP, with 25 µM dCTP and either 25 µM Cy3-labeled dCTP or Cy5-label dCTP. Microarray slides, pre-hybridized with the formamide based buffer Northern Max (Ambion) for 1–2 h at 42 °C, were further hybridized for 16–20 h at 42 °C with cDNA probes resuspended in 22 µl of the same buffer. After hybridization, slides were washed for removing excess probe and unspecific binding as described in [49]. Laser scanning of microarrays was performed using a ChipReader apparatus (Bio-Rad Laboratories, CA, USA) at 5 µm resolution. 16 bit TIFF images were analyzed by means of Genepix 6.0 software (Molecular Dynamics) to get raw fluorescence data from each spot. Three main microarray experiments were carried out. The first one consisted in gene expression profiling of digestive gland tissue of female individual mussels across 12 months (May 2007- April 2008) and included up to 40 microarray hybridizations. Dual color competitive hybridization analyses were based on a loop design in which each RNA obtained from month n was hybridized against that of month n+1. This design was performed in either three or four biological replicates using RNA samples obtained from single individual female animals from the most represented gonadal developmental stage. Relative mRNA abundances were further expressed respect to the values obtained in samples from January. The second microarray experiment consisted in gene expression profiling of digestive gland tissue of male and female individual mussels across the four gonadal developmental stages (Stage I, November 2007; Stage II, January 2008; Stage III, February, 2008; Stage IV March 2008). Dual color competitive hybridization analyses were carried out between RNA samples obtained from male and female single individuals. Four biological replicates were performed for a total of 16 microarray hybridizations. Relative mRNA abundances in male samples were expressed respect to the values obtained in female ones. The third microarray experiment consisted in gene expression profiling of mantle tissue in either male or female individual mussels across the four gonadal developmental stages (Stage I, November 2007; Stage II, January 2008; Stage III, February, 2008; Stage IV March 2008). Dual color competitive hybridization analyses were based on a loop design in which each RNA obtained from stage n was hybridized against that of stage n+1, with a total of 16 microarray hybridizations. This design was performed in four biological replicates using RNA samples obtained from single individuals. Relative mRNA abundances were further expressed respect to the values obtained in samples from resting stage (Stage I). Computational and statistical analysis of microarray data performed out using the Linear Mode for Microarray Analysis (LIMMA) software [50]. Offset background subtraction, loess normalization and least square regression were used along with moderated t-test and empirical Bayes statistics. A gene was considered statistically different in test condition versus the reference one for a log odd value (B) higher than 0. The whole procedure was carried out essentially as described in [51]. Miami compliant microarray data -including a detailed description of the experimental design and each hybridization experiment- were deposited in the Gene Expression Omnibus (GEO) database with the superSeries unique identifier GSE23052. The following link provide access to the deposited data http://www.ncbi.nlm.nih.gov/projects/geo/query/acc.cgi?acc = GSE23052.

### Q-PCR analysis

Q-PCR analyses were carried out from the same RNA extract used for microarray hybridization. Relative mRNA abundance levels of the mussel metallothionein genes mt10 (identified in the array with the EMBL ID AJ625847, see also Dataset S1) mt20 (which is not present in the array) and p53-like protein gene were evaluated through the SYBR green I chemistry, respectively according to [52] and [28] The mRNA abundance of chitinase genes AJ624093, AJ625569 and AJ624637 was evaluated in multiplex Taqman assay according to [51]. For other selected genes (AJ625256, matrilin isoform cra_b; AJ625621, hsp90; AJ625655, lethal giant larvae homolog 2; AJ624922, eukaryotic translation elongation factor 1 alpha 1; AJ624502, mam domain 2; AJ623584, nadh dehydrogenase subunit 5 and AJ516774, vitelline coat lysin m7) multiplex Taqman assay were set up ex novo. Probes and primer pairs were designed using the Beacon Designer v3.0 software (Premier Biosoft International, Inc.): all sequences are given in Table S1. cDNA (25 ng RNA reverse-transcribed to cDNA) was amplified into a CFX384 Realtime-PCR detection system (Bio-Rad laboratories) using the “iQTM Multiplex Powermix” (Bio-Rad laboratories) following the manufacturer instructions for the triplex protocol. All multiplex combinations accounted for the following dual fluorescence tags: 6-carboxyfluorescein (FAM)/Black Hole (BH)1; 6 - carboxy - 2′,4,4′,5′,7,7′ – hexachlorofluorescein *(*HEX)/BH1 and Texas Red/BH2. Briefly, cDNA was amplified in the presence of 1X iQTM Multiplex Powermix” (Bio-Rad laboratories), 0.3 µM each primer, and 0.1 µM each probe (Table S1) in a final volume of 10 µL. Relative expression data were geometrically normalized on 18S rRNA (L33452) and an invariant actin isotype (AJ625116). To this aim, a specific duplex Taqman assay was developed amplifying 0.25 ng RNA reverse-transcribed to cDNA in the presence of 0.1 µM each dual labelled probe (HEX/BH1 for actin; and Texas Red/BH2 for 18S rRNA), 0.1 µM and 0.4 µM each forward and reverse primer, respectively for 18S rRNA and actin (sequences are reported in Table S1).

For all Taqman assays, the thermal protocol was as follows: 30 s at 95 °C, followed by 40 cycles (10 s at 95 °C, 20 s at 60 °C). The Q-PCR reaction was performed on four biological replicates and three technical replicates. Statistical analysis were carried out on the group mean values using a random reallocation test [53]. All primers and dual labelled Taqman probes were synthesized by MWG-Biotech Gmbh (Germany).

### Functional genomic analysis

Functional characterization of mussel genes present in the array was based on GO annotation and carried out by means of the universal platform Blast2GO (B2GO) [54] using default parameters. Briefly, 1673 mussel sequence bearing a EMBL ID were subjected to the annotation analysis. 880 sequences showed no Blast-X [60] hits. Another 63 sequences showed no GO term mapping results. 873 mussel sequences were putatively annotated using GO terms obtained from the first 20 Blast-X hits or from protein domains obtained from InterProScan [61]. The latter results were reported in Dataset S1. GO term enrichment analysis was carried out through the implementation of a hypergeometric statistics (p<0.05) in which the distribution of GO terms in each set of interest was compared against the one relative the to whole microarray sequence catalogue. Cluster analysis of microarray data was computed using the Genesis software [55], [56].

## Supporting Information

Figure S1 **Q-PCR confirmation of the annual cycle gene expression trend (female digestive gland).** Shown are the average expression levels ± standard deviations relative to the reference condition (January) for the following genes: AJ624093, AJ625569, AJ624637, three different chitinases; AJ624922, eukaryotic translation elongation factor 1 alpha 1; AJ625256, matrilin isoform cra_b; AJ625243, p53-like protein gene; AJ625621, hsp90; AJ625847, mt10-IVb; AY566247, mt20; AJ625655, lethal giant larvae homolog 2; AJ624502, mam domain 2. All patterns, but that of p53-like could be confirmed. Data were geometrically normalized against actin and 18S rRNA. * Statistically different from the reference condition (January), p<0.05, random threshold cycle reallocation randomization test according to [53], n = 4.
(PDF)

Figure S2 **Q-PCR confirmation of the annual cycle gene transcriptomic trend (female mantle).** Shown are the average transcription levels ± standard deviations relative to the reference condition (Stage 1, early development) for the following genes: AJ625569, chitinase; AJ623584, nadh dehydrogenase subunit 5. Data were geometrically normalized against against actin and 18S rRNA. * Statistically different from the reference condition (January), p<0.05, random threshold cycle reallocation randomization test according to [53], n = 4
(PDF)

Figure S3 **Q-PCR confirmation of the annual cycle gene transcription trend (male mantle).** Shown are the average expression levels ± standard deviations relative to the reference condition (Stage 1, early development) for the following gene: AJ516774, acrosomal major protein M7. Data were geometrically normalized against actin and 18S rRNA. * Statistically different from the reference condition (January), p<0.05, random threshold cycle reallocation randomization test according to [53], n = 4.
(PDF)

Table S1 **Q-PCR primers and Taqman probes.**
(DOC)

Dataset S1 **Additional information to** **Fig. 2** **.** Output of the Gene Ontology term based gene annotation processes carried out using the bioinformatic platform Blast2GO.
(XLS)

Dataset S2 **Additional information to** **Fig. 3** **.** Gene Ids (EMBL) belonging to each k-means cluster.
(XLS)

Dataset S3 **Additional information to** **Table 2** **.** Differentially expressed genes in the digestive gland tissues of male vs female individuals (*Mytilus galloprovincialis*) sampled at stage 4 of gonad development. M represents the log2 relative expression of each gene in male vs female mussels. B represents the Bayes statistics. /represents mussel genes without an entry in the EMBL database.
(XLS)

Dataset S4 **Additional information to** **Fig. 4** **.** Log2 relative expression values (M) for the 369 differentially expressed genes (DEGs) identified in at least one condition across female gonad development (Stage 1 was used as reference condition). /represents mussel genes without an entry in the EMBL database.
(XLS)

Dataset S5 **Additional information to** **Fig. 5** **.** Log2 relative expression values (M) for the 354 differentially expressed genes (DEGs) identified in at least one condition across male gonad development (Stage 1 was used as reference condition). /represents mussel genes without an entry in the EMBL database.
(XLS)
