# Supplementary material for: Gene Expression Rhythms in the Mussel Mytilus galloprovincialis (Lam.) across an Annual Cycle
Source: PLoS One. 2011 May 5;6(5):e18904. doi: 10.1371/journal.pone.0018904 (PMC3088662; doi:10.1371/journal.pone.0018904)
Supplement: Figure S1 — Q-PCR confirmation of the annual cycle gene expression trend (female digestive gland). Shown are the average expression levels ± standard deviations relative to the reference condition (January) for the following genes: AJ624093, AJ625569, AJ624637, three different chitinases; AJ624922, eukaryotic translation elongation factor 1 alpha 1; AJ625256, matrilin isoform cra_b; AJ625243, p53-like protein gene; AJ625621, hsp90; AJ625847, mt10-IVb; AY566247, mt20; AJ625655, lethal giant larvae homolog 2; AJ624502, mam domain 2. All patterns, but that of p53-like could be confirmed. Data were geometrically normalized against actin and 18S rRNA. * Statistically different from the reference condition (January), p<0.05, random threshold cycle reallocation randomization test according to [53], n = 4. (PDF) [file pone.0018904.s001.pdf]

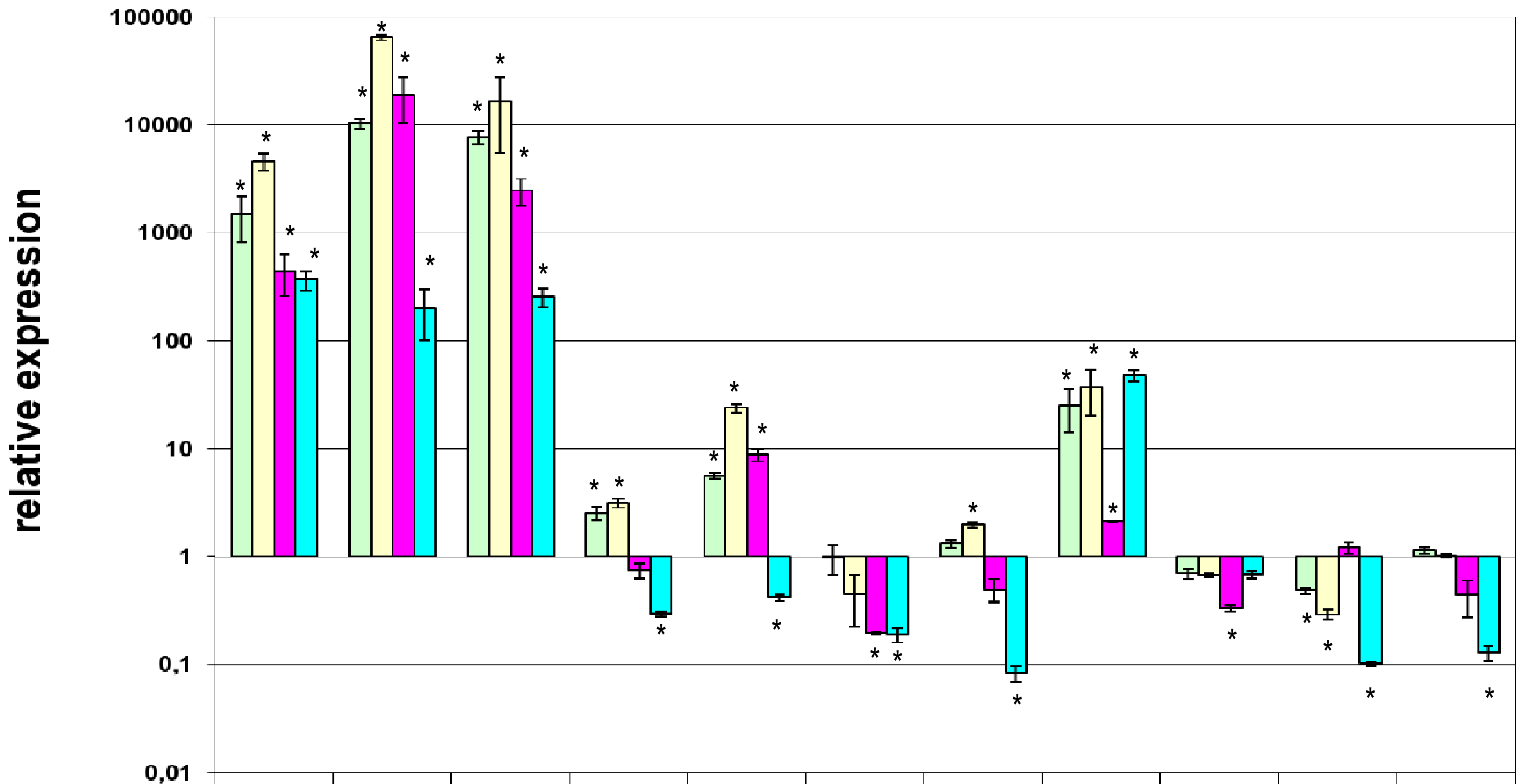

	AJ624093	AJ625569	AJ624637	AJ624922	AJ625256	AJ625243	AJ625621	AJ625847	AY566247	AJ624502	AJ625655
May	1506,31	10408,54	7740,19	2,53	5,61	0,98	1,32	25,09	0,69	0,48	1,14
June	4580,73	65491,71	16685,51	3,15	23,73	0,45	1,96	37,46	0,67	0,29	1,01
August	446,91	19004,44	2487,17	0,74	8,78	0,19	0,49	2,10	0,33	1,22	0,44
November	370,43	200,39	256,17	0,30	0,42	0,19	0,08	47,65	0,68	0,10	0,13

Suppl. Fig. S1. Q-PCR confirmation of the annual cycle gene expression trend (female digestive gland). Shown are the average expression levels ± standard deviations relative to the reference condition (January) for the following genes: AJ624093, AJ625569, AJ624637, three different chitinases; AJ624922, eukaryotic translation elongation factor 1 alpha 1; AJ625256, matrilin isoform cra_b; AJ625243, p53-like protein gene; AJ625621, hsp90; AJ625847, mt10-IVb; AY566247, mt20; AJ625655, lethal giant larvae homolog 2; AJ624502, mam domain 2. All patterns, but that of p53-like could be confirmed. Data were geometrically normalized against actin and 18S rRNA. * Statistically different from the reference condition (January), $p<0.05$, random threshold cycle reallocation randomization test according to [53], n=4
