# Supplementary material for: Gene Expression Rhythms in the Mussel Mytilus galloprovincialis (Lam.) across an Annual Cycle
Source: PLoS One. 2011 May 5;6(5):e18904. doi: 10.1371/journal.pone.0018904 (PMC3088662; doi:10.1371/journal.pone.0018904)
Supplement: Figure S3 — Q-PCR confirmation of the annual cycle gene transcription trend (male mantle). Shown are the average expression levels ± standard deviations relative to the reference condition (Stage 1, early development) for the following gene: AJ516774, acrosomal major protein M7. Data were geometrically normalized against actin and 18S rRNA. * Statistically different from the reference condition (January), p<0.05, random threshold cycle reallocation randomization test according to [53], n = 4. (PDF) [file pone.0018904.s003.pdf]

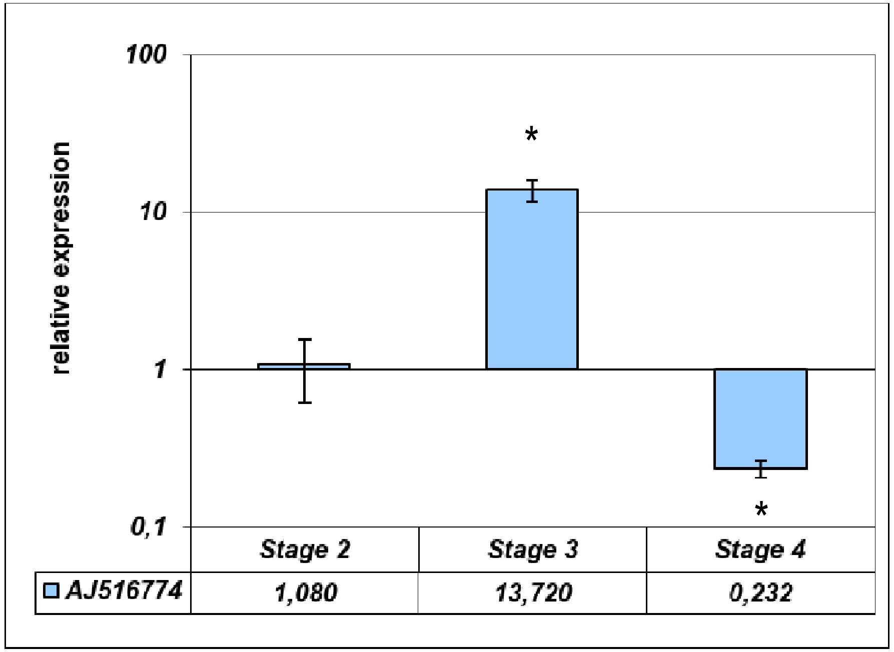

Suppl. Fig 3. Q-PCR confirmation of the annual cycle gene expression trend (male mantle). Shown are the average expression levels ± standard deviations relative to the reference condition (Stage 1, early development) for the following gene: AJ516774, acrosomal major protein M7. Data were geometrically normalized against actin and 18S rRNA. * Statistically different from the reference condition (January), $p<0.05$, random threshold cycle reallocation randomization test according to [53], n=4
