# Supplementary material for: Gene Expression Rhythms in the Mussel Mytilus galloprovincialis (Lam.) across an Annual Cycle
Source: PLoS One. 2011 May 5;6(5):e18904. doi: 10.1371/journal.pone.0018904 (PMC3088662; doi:10.1371/journal.pone.0018904)
Supplement: Table S1 — Q-PCR primers and Taqman probes. (DOC) [file pone.0018904.s004.doc]

Table S7. Q-PCR primers and Taqman probes

| # | Gene _ID | Probe | Sense Primer | Antisense Primer |
| --- | --- | --- | --- | --- |
| 1 | AJ625256 | AACTTTTCCACCACGCCCGCCATT | AACATATACAGGCAAAGCACTACA | TCACCATCCGTAATGATAATTGCA |
| 2 | AJ625621 | AAGTCCTCGCTTCCTCAGTCTCTCAACA | CACAGGTGAATCCAAAGATGTTGT | ACTCATCAATGGGGTCTATCATGT |
| 3 | AJ625655 | CTCCATGTGCCCTCTGAGTGAAACTTGT | TCAGTGATGATCCTAGATTAGGCA | CGTTCCTCTCTTTCCATCTGTAAC |
| 4 | AJ624922 | ACAAACTGTCGCCGTAGGAGTCATCAAA | CGTTTTGCTGTCCGAGACATG | CCACGCCTCACATCATTTCTTG |
| 5 | AJ624502 | AGAGGACCAACACCAACAACAATGACTG | AACGAGAACTTTGAATTCCAGAGA | CTCAGTAATCGGGCATTTTCCATA |
| 6 | AJ623584 | ACCCTTGTCACAGTCTTCTTAACCCACG | CTTATTGCACCTATTCTTACCGGC | TTCGTCTGTAAACTGTCCAACAAA |
| 7 | AJ516774 | TGAACCGTCAGGTTGTCCTGCTGACCA | ACACAGCGGCAAATGGGATT | TGCGTAGACTGCTAAGCATGTT |
| 8 | AJ625116 | ACGCCAACACCGTCTTGTCTGGTGG | GTGTGATGTCATATCCGTAAGGA | GCTTGGAGCAAGTGCTGTGA |
| 9 | L33452 | ACCACATCCAAGGAAGGCAGCAGGC | CGGAGAGGAGCATGAGAAAC | CGTGCCAGGAGTGGGTAATTT |

Given are: #, a progressive number; Gene ID, EMBL or NCBI gene Identifier; Taqman probe, sense primer and antisense primer sequences. All sequence are given 5' to 3'. Legend: AJ625256, matrilin isoform cra_b; AJ625621, hsp90; AJ625655, lethal giant larvae homolog 2; AJ624922, eukaryotic translation elongation factor 1 alpha 1; AJ624502, mam domain 2; AJ623584, nadh dehydrogenase subunit 5; AJ516774, vitelline coat lysin m7; AJ625116, actin; L33452, 18S ribosonal RNA
